# Progress in Surgery

**Published:** 1895-07-20

**Authors:** 


### Progress in Surgery.

### CEREBRAL SURGERY *(Continued from p. 258).*

Dr. Segmond brought before the Chirurgical Society of Paris[1] a case in which depressed fracture of the skull had not been recognised at the time of the injury and elevation was performed two months later with complete cure of the existing hemiplegia, for the depression lay over the motor area. The middle meningeal artery was wounded in the operation, and the opening had to be plugged with iodoform gauze, which had so closely adhered to the brain on the twelfth day, when it was removed, that a portion of brain substance had to be removed with it. Dr. Routier[2] has produced aphasia by pressure of iodoform gauze used to stop hæmorrhage, the symptoms passing off directly it was removed. Lucas-Championière recommends catgut for plugging in these cases, and Schwartz found it of service in plugging a wounded superior longitudinal sinus.

**Ligature of the Common Carotid Artery for Cerebral Hæmorrhage.**—Professor Horsley and Mr. Spencer have found that hæmorrhage in the basal ganglia can be controlled by ligature of the common carotid artery, and suggest that ligature of this artery might be employed in ingravescent apoplexy. Dr. Dercum and Dr. Keen report[3] two cases of ingravescent cerebral hæmorrhage treated by ligature of the common carotid artery. The first patient was a man aged 50, who on February 11th experienced first a weakness in the left arm and then in the left leg, and this condition of paresis increased on the following day, and again on the next day. By the 14th there was complete motor paralysis of the left arm, and distinct loss of power in the leg and of the face on the same side. There was no drowsiness. His heart and urine were normal. Dr. Keen, on the 14th, ligatured the common carotid under cocaine, as the effect of a general anæsthetic on the cerebral circulation was feared. The paralysis ceased to increase during the first two days after the operation, and then improvement set in, and in the course of two months became very considerable. The diagnosis was not by any means certain, but capsular hæmorrhage seemed more probable to the authors than embolism or thrombosis. In the second case the onset was too rapid to give ligature of the carotid any chance of doing good.

**Drainage of the Cerebro-Spinal Fluid.**—Fürbinger, at the Berlin Medical Society, read a paper on puncture of the spinal subarachnoid cavity—Quincke's puncture.[4] He makes the puncture with a small trochar or a Pravaz syringe, through the ligaments of the third and fourth lumbar arches, and allows the fluid slowly to drain away. He has punctured in this way 100 times, with no ill effects. Out of 37 cases of tuberculous meningitis tubercle bacilli were found in the fluid withdrawn in 30, the diagnosis being confirmed by autopsy in 27 of them. In one case of acute meningitis pneumococci were found. Withdrawal of the fluid relieved the pain in some cases of compression by cerebral tumour or from other causes. No improvement followed in several cases of basal meningitis in children, although sinking of the fontanelle showed that the intra-cranial pressure had been reduced. In some cases of uræmia or cerebral tumour sudden death followed the puncture. In cases of ventricular effusion of blood, spinal puncture revealed a large amount of blood in the spinal fluid; the amount was much less in cases of cortical hæmorrhage. The chemical character of the fluid was found to differ in cases of cerebral lesion and meningitis. In the former it contained (as usual) sugar, but not in the latter, in which condition much more albumen was present than in cases of cerebral tumour. These researches were carried out by Lichthein, who does not believe spinal puncture does any good in tuberculous meningitis, purulent meningitis, or cerebral tumour. He tried it in fifteen such cases. Fraenkel has noticed diminution in the swelling of the optic disc and relief of other symptoms after it. Dr. Browning, of New York, contributes a paper on lumbar puncture of the cerebrospinal space of the cord.[5] He thinks it can only give temporary amelioration in cases of cerebral tumour and meningitis. The author records a case of increasing hydrocephalus, in which the condition of the child was not influenced by the operation and death occurred. He thinks it is best to make the puncture between the third and fourth lumbar vertebræ, and considers that it cannot often be done between the fourth and fifth. The trochar (an aspirating needle does well) should be entered a little to one side of the middle line, though in children he thinks it better to pass it directly between the spinous processes, tilting it up a little. On withdrawing his tube iodoform collodion is applied. Neither in hydrocephalus or tuberculous meningitis does he consider the method likely to be of benefit. Frank[6] thinks that drainage of the lateral ventricles is clearly indicated in cases of acute, simple, or tuberculous meningitis, and is likely to be useful in chronic hydrocephalus with moderate distension of the ventricles, without enlargement of the head, and that it makes recovery possible in ventricular hæmorrhage.

**Operation for Spasmodic Wryneck.**—Drs. Richardson and Walton, of Massachusetts,[7] consider that treatment of spasmodic torticollis by drugs, apparatus, or electricity will rarely prove successful in well-established cases; but that massage may prove of value in comparatively recent cases. Resection of nerves, they think, affords practically the only rational remedy. In cases of spasm of the sterno-mastoid and trapezius the spinal accessory nerve is resected; in spasmodic contraction of the posterior rotator muscles the posterior cervical roots are divided, as recommended by Noble Smith in this country and Keen in America. The authors consider that operation on the spinal accessory nerve for sterno-mastoid spasm may afford relief, even if other muscles are affected, but that spasm previously limited to the sterno-mastoid may spread to other muscles in spite of the operation. They regard spasm of the sterno-mastoid on one side and of the posterior rotators on the other (which would turn the head towards the same side, *i.e.*, the side to which also the sterno-mastoid contraction would incline it) as the most common combination of spasm. In cases in which the posterior roots are divided we need not fear inability to hold the head erect. References are given to descriptions of the operation on the posterior nerve roots by Keen and Noble Smith, and several cases are reported of resection of the spinal accessory nerve. They exposed the nerve from the anterior border of the sterno-mastoid just as it is about to pierce the muscle. Irritation of the tissues in the floor of the wound with a blunt instrument often enabled them to discover its presence, by causing contraction of the muscles supplied by it. The cases seem to show that great relief may be obtained by resection of the spinal accessory nerve in spasm of the sterno-mastoid, though it may be only temporary in some cases, and that even when spasm in the muscle has ceased as a result of such resection, still other muscles may subsequently become affected by the spasm. In one case of bilateral spasm of the posterior rotators, massage and stretching, practised three times a week for seven weeks, cured the patient. This was a recent case, and is not an exception, the authors consider, to the dictum of Noble Smith that treatment other than operation is ineffectual in well-established cases of spasmodic torticollis.

**Craniectomy in Microcephalic Idiots.**—The question as to the advisability of operating on microcephalic skulls is still much discussed. Dr. Jacobi showed that only when there was evidence of premature synostosis could the operation be expected to do good, but the evidence of this condition was not satisfactory unless we had proof of too early closures of the fontanelles and premature eruption of the teeth, occurring in the upper jaw first instead of the lower. The mortality of the operation in these infants, from shock, he found to be very great. Professor Humphreys has examined nineteen specimens of idiot skulls,[8] and considers that there is nothing to suggest that the deficiency in the development of the skull was the cause of the idiocy by exerting a compressing influence on the brain, and therefore that craniectomy in such cases is not a rational procedure. Dr. Wallis Ord and Mr. Edward Cotterell record[9] a case of what they considered to be microcephalic idiocy treated by craniectomy with distinct improvement. The infant at sixteen months had very severe general convulsions, after which general loss of power and impairment of intelligence set in. The normal relation in size between the face and the skull was reversed, and the fontanelles were considered prematurely closed. When nineteen months old the operation was performed in sections. After the second operation improvement began, and was steadily maintained until she became like an ordinary child. Four operations were performed. Dr. Shuttleworth[10] has had under his care three microcephalic idiots which had not improved at all after operation. He considered that in such cases there was an arrest of development of the brain at the fifth or sixth month of fœtal life, and the premature union of the bones was secondary to the arrest of cerebral growth. Beck, from a statistical study[11] of cases of microcephalic idiots treated by craniectomy, finds that the mortality in 72 reported cases is 17 per cent., and that the dangers of operation are shock, hæmorrhage, and infection. Six of the 12 fatal cases died of shock, one of hæmorrhage, and two of septic infection. He advises that the chisel and hammer should be discarded, in order to avoid shock, and thinks that bleeding from the skull can be checked by Esmarch's bandage applied round the head. Binnie[12] advises craniectomy in cases of non-microcephalic idiocy, and records two cases, one of which was fatal. On what grounds he performs such an operation he fails to make clear. Dr. Seneca D. Powell[13] has operated on 14 cases of microcephalic idiots and only lost one patient. The ages of the patients varied from two to thirteen years. He removed a strip of bone five inches long and half an inch wide between the trephine holes, with a saw worked with an electromotor. Hæmorrhage was checked by the application of a bandage and a strip of adhesive rubbed plaster around the head. Dr. Dana[14] considered the low mortality which Dr. Powell had secured very surprising and gratifying, but put forward the view of Professor Humphry and Dr. Shuttleworth, that the premature ossification was secondary to imperfect brain development, and that the child was not likely to be benefited by craniectomy.

1 Medical Week, 1894, June 15th, p. 283. 2 Ibid. 3 The Journal of Nervous and Mental Diseases, Sept., 1894, p. 586. 4 Berl. Klin. Woch., 1895, No 13; Epitome Brit. Med. J., April 27th, 1895; and Lancet, April 20th, 1895. 5 The Journal of Nervous and Mental Diseases, Oct., 1894. 6 Annals of Surgery, April, 1894, and Epitome Brit. Med. J., June 30th, 1894. 7 Amer. Journ. Med. Sci., Jan., 1895, p. 27. 8 Lancet, Feb. 16th, 1895, and Journ. Anat. and Phys. 9 Lancet, March 2nd, 1895. 10 Ibid. 11 Journ. of Amer. Med Assoc., Nov. 3rd, 1894, and Therap. Gaz., Dec. 15th, 1894. 12 Annals of Surgery, April, 1894, p. 453. 13 Med. Record, April 13th, 1895, p. 477. 14 Ibid.

